# Assessment of anxiety and depression in patients with incidental pulmonary nodules and analysis of its related impact factors

**DOI:** 10.1111/1759-7714.13406

**Published:** 2020-03-25

**Authors:** Lihong Li, Yan Zhao, Hui Li

**Affiliations:** ^1^ Department of Thoracic Surgery, Beijing Chaoyang Hospital Capital Medical University Beijing China

**Keywords:** Anxiety, depression, pulmonary nodules, relevance, social support

## Abstract

**Objectives:**

To assess anxiety and depression in patients with incidental pulmonary nodules and analyze the related impact factors.

**Methods:**

All patients were assessed by questionnaires for their anxiety and depression after incidentally found pulmonary nodules. Hospital anxiety and depression scale (HAD), generalized anxiety disorder scale‐7 (GAD‐7), and multidimensional scale of perceived social support (MSPSS) were used to evaluate their anxiety and depression in order to understand the basic information and social support, and to analyze the related factors.

**Results:**

The HAD scale was used in 201 patients with pulmonary nodules. Univariate analysis showed the frequency of visits and social support had significant effects on anxiety (χ^2^ = 9.604, 20.912, *P* < 0.05). Regression analysis showed that social support (OR = 4.042, 95% CI: 2.1617.558, *P* < 0.05) was an independent influencing factor of anxiety. Univariate analysis showed that marital status, exposure history and social support had significant effects on depression (χ^2^ = 10.626, 6.005, 3.984, *P* < 0.05). Regression analysis showed that marital status (OR = 0.375, 95% CI: 0.186–0.754, *P* < 0.05) and social support (OR = 2.206, 95% CI: 1.016–4.789, *P* < 0.05) were independent influencing factors of depression. The results of GAD‐7 showed the incidence of anxiety in patients with pulmonary nodules was 59.3% (108/182). Univariate analysis showed that anxiety was correlated with a history of chronic disease, psychological disease and social support (χ^2^ = 9.949, 8.356, 11.872, *P* < 0.05). Further regression analysis showed that a previous history of psychological disease (OR = 5.088, 95% CI: 1.804–14.339) and social support (OR = 2.768, 95% CI: 1.505–5.094), were independent influencing factors of anxiety.

**Conclusions:**

The results of the study concluded that anxiety was affected by social support and previous psychological factors, while depression was affected by marital status and social support. Doctors should therefore strengthen communication with patients, and alleviate the negative emotions of patients as far as possible.

## Introduction

Pulmonary nodules are circular lesions with a diameter ≤3 cm on computed tomography (CT) scan, which can be solitary or multiple, without atelectasis, hilar lymph node enlargement and pleural effusion.^1^ In recent years, with the increased attention to routine physical examination and popularity of low‐dose spiral CT scan, the number of patients with new incidental pulmonary nodules has gradually increased.^2^ According to a recent study, nearly 26.3*%* of pulmonary nodules were found on annual routine checkup,^3^ most of which were benign.^4^ Although the probability of malignant pulmonary nodules was generally low, due to the asymmetry of patient information and fear of lung cancer, some were always in a state of anxiety and even depression after finding pulmonary nodules. This psychological status not only affects the physical and mental health of patients, but also reduces their quality of life.^5^ Furthermore, if they have psychological distress before surgery, it will not only increase the incidence of postoperative adverse events and the cost of medical services, but also cause mental stress and fatigue in the family members who are responsible for taking care of the patients.^6^ However, there are few studies on the psychological issues in patients with incidental pulmonary nodules. Therefore, the purpose of the current study was to assess the anxiety and depression status in patients with incidental pulmonary nodules, analyze the related impact factors and provide helpful information for medical staff in order to identify high‐risk patients before commencement of clinical evaluation.

## Methods

### Patients

Between February 2019 and July 2019, 201 consecutive patients with incidental pulmonary nodule lesions seen at the Department of Thoracic Surgery, Beijing Chaoyang Hospital, were investigated via completion of a questionnaire.

Inclusion criteria were defined as follows: (i) patients were >18‐years‐old; (ii) had good communication; (iii) no visual and hearing impairment; (iv) were aware of their primary disease; (v) chest CT scan showed pulmonary nodules with a diameter of ≤3 cm, which had not yet been identified as benign or malignant; and (vi) the patient or their family member agreed to complete the questionnaire and sign the informed consent form.

Exclusion criterion were as follows: (i) patients were <18‐years‐old; (ii) had visual and hearing impairment; (iii) did not know their primary disease; (iv) chest CT scan showed a lung mass with a diameter >3 cm, or which had been confirmed to be benign or malignant and the patient was aware of the diagnosis; (v) the patient had a language communication disorder; (vi) cerebrovascular sequela limb paralysis; and (vii) the patient or their family member refused to complete the questionnaire, or sign the informed consent form.

The questionnaire was completed electronically via the internet (http://www.wenjuan.com). The testers were trained in advance to master unified instructions and procedures. First, the purpose and method of the survey were explained to patients during the investigation. After obtaining informed consent and signing the consent form, the QR code of the questionnaire was presented to the patients for completion. If there were any difficulties in communication with the patients, then the questionnaire could be completed by the accompanying family member. To avoid duplicate answers or multiple submissions by the same person, each computer or cell phone (ie, each ip address) could be submitted only once.

### Questionnaire

The questionnaire consisted of four parts. The first part contained both clinical and social data of patients, including gender, age, educational experience, marital status, smoking history, chronic disease history, malignant tumor history, family history, symptoms, profession, the first visit or second visit, exposure history of smoking, radioactive substances and minerals, and history of mental illness.

In the second part, the anxiety and depression states of the patients were evaluated by the hospital anxiety and depression scale (HAD).^7^ The HAD was composed of 14 items, of which seven items were depression relevant and seven items were anxiety relevant. Letter “A” represented anxiety items and “D” depression items. Each item was divided into four grades (grades 0, 1, 2 and 3). The total scores were obtained by superimposing the two items separately. The positive detection criteria of anxiety and depression were all ≥8 points.

In the third part, the generalized anxiety disorder scale‐7 (GAD‐7) was used to evaluate the anxiety state of patients.^8^ The score of each item was 0–3, and the total score was the sum of the scores of each item. Among these, 0–4 had no anxiety, 5–10 had mild anxiety, 11–14 had moderate anxiety, and 15–21 had severe anxiety.

In the fourth part, the multidimensional scale of perceived social support (MSPSS) was used to evaluate the social relationships of patients.^9^ It emphasized individual self‐understanding and self‐feeling. The degree of social support from family and friends and others was measured separately, and the total score was used to reflect the total degree of social support. MSPSS was divided into three categories: family support, friend support and other support. The scale contained 12 self‐rating items, using the seven‐level scoring method, the total score of social support was accumulated by all items, and the total score reflected the total degree of social support. The total score between 12 and 36 was defined as low support state, 37–60 as intermediate support state, and 61–84 as high support state. The higher the total score, the higher the individual's social support.

### Statistical analysis

The Statistical Package for Social Science software (version 22.0) was used for statistical analysis. The normally distributed measurement data were expressed by mean and standard deviation ($\bar{X\;}$± s) and the others by median and associated interquartile range M (P25, P75), and the counting data were described by frequency and percentage. In univariate analysis, chi‐square test or Fisher's exact probability method was used to analyze the counting data, Kruskal‐Wallis rank sum test was used to analyze the grade data, and logistic regression analysis was used in multivariate analysis. *P*‐value <0.05 indicated statistical significance.

## Results

### Basic patient data

A total of 201 patients with incidental pulmonary nodules were enrolled in this study, including 201 patients who were evaluated with HAD and 183 patients with GAD‐7. There were 84 males (41.8*%*) and 117 females (58.2*%*) in the study. A total of 29 cases (14.4*%*) were <40‐years‐old, 108 (53.6*%*) were 40–60‐years‐old, and 64 (32.0*%*) were >60‐years‐old. Marriage statuses were unmarried 10 (5.0%), married 176 (87.6*%*), widowed seven (3.4*%*), and divorced eight (4.0*%*). There were 122 patients who received surgery, of which 15 cases (12.2%) had benign nodules and 107 cases (87.8%) had malignant nodules. The average size of pulmonary nodules was 12.47 ± 5.84 mm. There was no significant difference in the size of pulmonary nodules between the anxiety or the depression group (see Table 1). The other 79 patients were followed up regularly without surgery.

**Table 1:** Demographic and medical characteristics (n[%])

	Number of patients (%)	
Item	HAD	GAD‐7
Gender		
Male	84 (41.8%)	72 (42.0%)
Female	117 (58.2%)	111 (58.0%)
Age		
<40	29 (14.4%)	27 (14.7%)
>60	64 (32.0%)	61 (33.3%)
40–60	108 (53.6%)	95 (52.0%)
Educational experience		
Elementary school and below	10 (5.0%)	9 (4.9%)
Junior high school	45 (22.4%)	43 (23.5%)
High school and secondary school	52 (25.9%)	47 (25.7%)
College and undergraduate	83 (41.3%)	74 (40.4%)
Graduate and above	11 (4.9%)	10 (5.5%)
Marital status		
Unmarried	10 (5.0%)	9 (4.9%)
Married	176 (87.6%)	161 (88.0%)
Widowed	7 (3.4%)	6 (3.3%)
Divorce	8 (4.0%)	7 (3.8%)
History of occupational exposure		
Yes	39 (19.4%)	35 (19.1%)
No	162 (80.6%)	148 (80.9%)
History of smoking		
Yes	66 (32.9%)	56 (30.6%)
No	135 (67.1%)	127 (69.4%)
Chronic disease history		
Yes	54 (26.9%)	50 (27.3%)
No	147 (73.1%)	133 (72.7%)
History of malignant tumor		
Yes	11 (4.9%)	11 (6.0%)
No	190 (95.1%)	172 (94.0%)
Family history		
Yes	47 (23.4%)	43 (23.5%)
No	154 (76.6%)	140 (76.5%)
Is there a symptom?		
Yes	156 (77.6%)	138 (75.4%)
No	45 (22.4%)	45 (24.6%)
Profession		
National public servant	23 (11.4%)	22 (12.0%)
Peasant	16 (8.0%)	15 (8.2%)
Enterprise managers	44 (21.9%)	37 (20.2%)
Freelancer	25 (12.4%)	21 (11.5%)
Teacher	14 (7.0%)	13 (7.1%)
Medical worker	8 (4.0%)	8 (4.4%)
Other	71 (35.3%)	67 (36.6%)
Whether the first diagnosis		
First diagnosis	132 (65.7%)	121 (66.1%)
Subsequent visit	69 (34.3%)	62 (33.9%)
History of mental illness		
Yes	15 (7.5%)	14 (7.7%)
No	186 (92.5%)	169 (92.3%)
Social support		
Moderate social support	80 (39.8%)	65 (35.5%)
High social support	121 (60.2%)	117 (64.5%)

### Analysis of impact factors of anxiety status by HAD scale in patients with pulmonary nodules

The results of this study showed that the size of nodules had no statistical significance in patients with anxiety and depression. A total of 80 cases (39.8*%*) had intermediate support, 121 cases (60.2*%*) had high support, and there was no patient with low support status. HAD scale showed anxiety in 64 cases (31.8*%*) and nonanxiety in 137 cases (68.2*%*). The results of univariate analysis of anxiety status of HAD scale in patients with incidental pulmonary nodules showed that the number of clinic visits (X^2^ = 9.604, *P* < 0.05) and social support (X^2^ = 20.912, *P* < 0.01) had significant effects on anxiety in patients with pulmonary nodules (see Table 2). Further regression analysis showed that patients with intermediate support were more likely to have anxiety than patients with high support (see Table 3).

**Table 2:** Analysis of anxiety influencing factors of HAD scale in patients with pulmonary nodules (n[%])

Item	Anxiety	Nonanxiety	X^2^ value	*P*‐value
Gender			0.006	0.938
Male	27 (13.4%)	57 (28.4%)		
Female	37 (18.4%)	80 (39.8%)		
Age			0.035	0.983
<40	9 (4.5%)	20 (10%)		
>60	20 (10%)	44 (21.9%)		
40–60	35 (17.4%)	73 (36.2%)		
Educational experience			1.097	0.895^a^
Elementary school and below	3 (1.5%)	7 (3.5%)		
Junior high school	14 (7%)	31 (15.4%)		
High school and secondary school	17 (8.5%)	35 (17.4%)		
College and undergraduate	25 (12.4)	58 (28.9%)		
Graduate and above	5 (2.5%)	6 (2.9%)		
Marital status			0.168	0.983^a^
Unmarried	3 (1.5%)	7 (3.5%)		
Married	56 (27.8%)	120 (59.8%)		
Widowed	2 (1%)	5 (2.5%)		
Divorce	3 (1.5%)	5 (2.5%)		
History of occupational exposure			0.977	0.323
Yes	15 (7.5%)	24 (12%)		
No	49 (24.3%)	113 (56.2%)		
History of smoking			1.651	0.199
Yes	25 (12.4)	41 (20.4%)		
No	39 (19.4%)	96 (47.2%)		
Chronic disease history			1.690	0.194
Yes	21 (10.4%)	33 (16.4%)		
No	43 (21.4%)	104 (51.8%)		
History of malignant tumor			1.168	0.184^a^
Yes	6 (3%)	5 (2.5%)		
No	58 (28.9%)	132 (65.6%)		
Family history			0.137	0.711
Yes	16 (8%)	31 (15.4%)		
No	48 (23.9%)	106 (52.7%)		
Is there a symptom?			0.233	0.629
Yes	51 (25.4%)	105 (52.2%)		
No	13 (6.5%)	32 (15.9%)		
Profession			3.411	0.756^a^
Whether the first diagnosis			0.419	0.517
First diagnosis	40 (19.9%)	92 (45.8%)		
Subsequent visit	24 (11.9%)	45 (22.4%)		
Number of visits			9.604	0.048^a^
History of mental illness			3.772	0.152^a^
Yes	5 (2.5%)	10 (5%)		
No	59 (29.3%)	127 (63.2%)		
Social support			20.192	0.000
Moderate social support	40 (19.9%)	40 (19.9%)		
High social support	24 (12%)	97 (48.3%)		

Note: A minimum theoretical frequency <5 with Fisher's exact probability method.

**Table 3:** Logistic regression analysis of influencing factors of HAD scale in patients with pulmonary nodules

Item	OR (95% CI)	*P*‐value
HAD‐A		
Social support		
Moderate social support	4.042 (2.161–7.558)	<0.05
High social support	1.00	
HAD‐D		
Marital status	0.375 (0.186–0.754)	<0.05
Social support		
Moderate social support	2.206 (1.016–4.789)	<0.05
High social support	1.00	

Note: HAD‐A stands for anxiety status; HAD‐D stands for depression status.

### Analysis of influencing factors of depression status by HAD scale in patients with pulmonary nodules

HAD showed depression in 39 cases (19.4*%*) and no depression in 162 cases (80.6*%*). Univariate analysis of depression status by HAD scale in patients with pulmonary nodules showed that marital status (X^2^ = 10.626, *P* < 0.05), occupational exposure history (X^2^ = 6.005, *P* < 0.05), and the degree of social support (X^2^ = 3.984, *P* < 0.05) had a statistically significant effect on depression in patients with pulmonary nodules (see Table 4). Further regression analysis showed that being widowed, divorced and intermediate support were risk factors for depression in patients with pulmonary nodules (see Table 3).

**Table 4:** Analysis of depression influencing factors of HAD scale in patients with pulmonary nodules (n[%])

Item	Depression	Nondepressed	X^2^ value	*P*‐value
Gender			0.221	0.639
Male	15 (7.5%)	69 (34.3%)		
Female	24 (11.9%)	93 (46.3%)		
Age			4.623	0.099
<40	4 (2%)	25 (12.4%)		
>60	8 (4%)	46 (22.9%)		
40–40	17 (8.4%)	91 (45.3)		
Educational experience			2.563	0.633^a^
Elementary school and below	1 (0.5%)	9 (4.5%)		
Junior high school	7 (3.5%)	38 (18.9%)		
High school and secondary school	13 (6.5%)	39 (19.4%)		
College and undergraduate	15 (7.5%)	68 (33.8%)		
Graduate and above	3 (1.5%)	8 (4%)		
Marital status			10.626	0.014^a^
Unmarried	0 (0%)	10 (5%)		
Married	32 (16%)	144 (71.7%)		
Widowed	4 (2%)	3 (1.5%)		
Divorce	3 (1.5%)	5 (2.5%)		
History of occupational exposure			6.005	0.014
Yes	13 (6.5%)	26 (12.9%)		
No	26 (12.9%)	136 (67.7%)		
History of smoking			0.005	0.941
Yes	13 (6.5%)	53 (26.4%)		
No	26 (12.9%)	109 (54.2%)		
Chronic disease history			2.009	0.156
Yes	14 (7%)	40 (19.9%)		
No	25 (12.4%)	122 (40.6%)		
History of malignant tumor			1.147	0.284^a^
Yes	4 (2%)	7 (3.5%)		
No	35 (17.4%)	155 (77.1%)		
Family history			1.474	0.225
Yes	12 (6%)	35 (17.4%)		
No	27 (13.4%)	127 (63.2%)		
Is there a symptom?			0.549	0.459
Yes	32 (15.9%)	124 (61.7%)		
No	7 (3.5%)	38 (18.9%)		
Profession			10.742	0.097^a^
Whether the first diagnosis			0.053	0.818
First diagnosis	25 (12.5%)	107 (53.2%)		
Subsequent visit	14 (7%)	55 (27.3%)		
Number of visits			6.778	0.148^a^
History of mental illness			0.547	0.460
Yes	4 (2%)	11 (5.5%)		
No	35 (17.4%)	151 (75.1%)		
Social support			3.984	0.046
Moderate social support	21 (10.4%)	59 (29.4%)		
High social support	18 (9%)	103 (51.2%)		

Note: A minimum theoretical frequency <5 with Fisher's exact probability method.

### Analysis of influencing factors of anxiety status by GAD‐7 scale in patients with pulmonary nodules

A total of 182 patients were evaluated by GAD‐7 scale. The scale showed 74 (40.7*%*) nonanxious patients, and 108 (59.3*%*) with anxiety. Among these, there were 73 (40.1*%*) with mild anxiety, 20 (11.0*%*) with moderate anxiety and 15 (8.2*%*) with severe anxiety. Univariate analysis of anxiety status by GAD‐7 scale in patients with pulmonary nodules showed that history of chronic diseases (X^2^ = 9.949, *P* < 0.05), psychological diseases (X^2^ = 8.356, *P* < 0.05) and the degree of social support (X^2^ = 11.872, *P* < 0.05) had statistically significant effects on anxiety in patients with pulmonary nodules (see Table 5). Further regression analysis showed that history of psychological disease and moderate social support were the risk factors which affected the anxiety of patients with pulmonary nodules (see Table 6).

**Table 5:** Analysis of influencing factors of anxiety status of GAD scale in patients with pulmonary nodules [n(%)]

		Anxiety				
Item	No anxiety	Mild	Moderate	Severe	H value	*P* value
Gender					3.396	0.335
Male	34 (18.7%)	25 (13.7%)	9 (4.5%)	4 (2.0%)		
Female	40 (22%)	48 (26.4%)	11 (6.0%)	11 (6.0%)		
Age					5.535	0.137
<40	7 (4.0%)	16 (9.0%)	3 (2.0%)	1 (0.5%)		
>60	25 (13.7%)	25 (13.7)	7 (3.8%)	4 (2.2%)		
40–60	42 (23.0%)	32 (17.6%)	10 (5.5%)	10 (5.5%)		
Educational experience					1.492	0.684
Elementary school and below	3 (1.5%)	4 (2.2%)	1 (0.5%)	1 (0.5%)		
Junior high school	18 (9.9%)	21 (11.5%)	1 (0.5%)	3 (2.0%)		
High school and secondary school	21 (11.5%)	14 (7.7%)	8 (4.3%)	3 (2.0%)		
College and undergraduate	29(16.0%)	29 (16.0%)	8 (4.3%)	8 (4.3%)		
Graduate and above	3 (1.5%)	5 (2.7%)	2 (1.0%)	0 (0%)		
Marital status					2.486	0.478
Unmarried	2 (1.1%)	6 (3.3%)	0 (0%)	1 (0.5%)		
Married	68 (37.0%)	61 (33.5%)	18 (10%)	14 (7.7%)		
Widowed	1 (0.5%)	5 (2.7%)	0 (0%)	0 (0%)		
Divorce	3 (2.0%)	1 (0.5%)	2 (1.1%)	0 (0%)		
History of occupational exposure					7.807	0.050
Yes	15 (8.2%)	9 (4.9%)	8 (4.3%)	3 (1.5%)		
No	59 (32.4%)	64 (35.2%)	12 (6.6.%)	12 (6.6.%)		
History of smoking					3.350	0.341
Yes	26 (14.3%)	20 (11.0%)	7 (3.8%)	2 (1.1%)		
No	48 (26.4%)	53 (29.1%)	13 (7.1%)	13 (7.1%)		
Chronic disease history					9.949	0.019
Yes	19 (10.4%)	15 (8.2%)	6 (3.3.%)	9 (4.9%)		
No	55 (30.2%)	58 (31.8%)	14 (7.7%)	6 (3.3%)		
History of malignant tumor					3.061	0.382
Yes	5 (2.7%)	2 (1.1%)	1 (0.5%)	2 (1.1%)		
No	69 (37.9%)	71 (39.0%)	19 (10.4%)	13 (7.1%)		
Family history					1.784	0.618
Yes	16 (8.8.%)	16 (8.8%)	7 (3.8%)	43 (23.6%)		
No	58 (31.9%)	57 (31.3%)	13 (7.1%)	11 (6.0%)		
Is there a symptom?					1.922	0.589
Yes	56 (30.8%)	52 (28.5%)	16 (8.8%)	13 (7.1%)		
No	18 (9.9%)	21 (11.5%)	4 (2.2%)	2 (1.1%)		
Profession					5.281	0.152
Whether the first diagnosis?					0.734	0.865
First diagnosis	50 (27.4%)	46 (25.3%)	13 (7.1%)	11 (6.0%)		
Subsequent visit	24 (13.2%)	27 (14.8%)	7 (3.8%)	4 (2.2%)		
Number of visits					1.357	0.716
History of mental illness					8.356	0.039
Yes	3 (2.0%)	5 (2.7%)	1 (0.5%)	5 (2.7%)		
No	71 (39.0%)	68 (37.3%)	19 (10.4%)	30 (16.5%)		
Social support					11.872	0.008
Moderate social support	17 (9.3%)	30 (16.5%)	11 (6.0%)	7 (3.8%)		
High social support	57 (31.3%)	43 (23.6%)	9 (4.9%)	8 (4.4%)		

**Table 6:** Logistic regression analysis of influencing factors of anxiety status of GAD scale in patients with pulmonary nodules

Item	OR (95% CI)	*P*‐value
History of mental illness		
Yes	5.088 (1.804–14.339)	<0.05
No	1.00	
Social support		
Moderate social support	2.768 (1.505–5.094)	<0.05
High social support	1.00	

## Discussion

With the continuous progress of medical technology, modern medicine has changed from the “biomedical model” to “biology, psychology and social medical model”. It means that doctors should pay more attention to the psychological rehabilitation of patients while treating the disease. In recent years, the detection rate of pulmonary nodules has increased annually. It has been reported that some patients have psychological problems after being told they have pulmonary nodules.^10^ Among these psychological problems, the most common are anxiety and depression. Anxiety is a feeling of inner nervousness when stressed, and depression is a negative emotion characterized by low mood and reduced interest. Both of these belong to the defensive psychological reaction of the human body, and these two kinds of psychological reaction are also called pathological emotional response.^11^ Bad psychological emotions not only affect the physical and mental health of patients, but also have disadvantages for family members. and even society. However, at present, there is still a lack of attention to psychological anxiety and depression in patients with incidental pulmonary nodules in China.^12^

Some studies have confirmed that anxiety and depression can inhibit the immune function of patients, reduce their pain threshold, increase the risk of tumor deterioration, lead to a decline in quality of life, and reduce their expected survival by almost 10% to 20%, They may even lead to the direct death of some patients, and the reason may not be the continuous deterioration of the disease, but because they are anxious and depressed.^13^ If preoperative patients suffer from psychological distress before surgery, it may lead to the decline of postoperative cognitive function and aggravation of pain, the incidence of postoperative adverse events and correspondingly to an increase in the investment of medical services. It can also cause mental stress and fatigue among the family members who are responsible for taking care of the patients, and affect the quality of life of the patients and their families.^14^ Some studies have found that anxiety and depression in surgical patients during the preoperative period will bring many adverse effects and compromise surgical outcomes. Anxiety can increase the excitability of the sympathetic nervous system, thus increasing the dosage of anesthetics and analgesics after post operation, prolonging extubation time, delaying anaesthetic recovery and increasing adverse reactions such as nausea, vomiting and shivering.^15^ In addition, depression can reduce the stress ability of patients and increase the incidence of postoperative infection.^16^ Therefore, while treating the disease, doctors should determine the psychological problems of the patients in time and correct their negative emotion as soon as possible.

As for anxiety and depression in patients, previous studies have been conducted in different diseases and different populations. Studies have shown that patients with coronary heart disease, asthma, chest pain and other diseases may have obvious anxiety and depression problems.^17^, ^18^, ^19^ Anxiety is associated with sex, age, pain intensity and pain treatment, while depression is associated with gender, pain intensity and duration of treatment, according to a study of psychological problems in patients with chronic pain.^20^ The results of a survey of veterans reported that the incidence of anxiety in patients with pulmonary nodules was 39%, and that anxiety and depression are related to the quality of communication. High‐quality communication can reduce the perceived risk of patients, and reduce the degree of anxiety and depression in patients.^21^

In this study, HAD and GAD‐7 were used to evaluate the state of anxiety and depression in patients with pulmonary nodules. HAD has good reliability and validity, and meets the requirements of psychometry. Because of its simplicity, it has been widely used in the evaluation of anxiety and depression in patients with various clinical diseases. Previous studies have found that Cronbach's α coefficients of the total subscale, anxiety subscale and depression subscale of HAD were 0.879, 0.806 and 0.806, respectively. It was previously considered that a Cronbach's α coefficient greater than 0.6 indicated good homogeneity. Therefore, the scale can be used as a screening tool for anxiety and depression in general hospitals.^22^ GAD‐7 can not only be used to screen GAD, but also to evaluate the severity of the disease according to the score. It has been proved that GAD‐7 had good reliability and validity.^23^ A medical center in Germany used HAD and GAD‐7 scales to investigate the diagnostic accuracy of anxiety in 2141 cancer patients. The study by Esser *et al*. demonstrated that GAD‐7 and HADS‐A had sufficient diagnostic accuracy and were therefore suitable for GAD screening in cancer patients.^24^ In this study, the HAD scale showed that the incidence of anxiety was 31.3%, depression was 19.4%, and the GAD‐7 scale showed that the incidence of anxiety was 59.3%, indicating that anxiety and depression were common among patients with lung nodules, and is consistent with Christopher's findings. The anxiety of patients was affected by the degree of social support and history of psychological diseases. There was a significant increase in the incidence of anxiety among patients with intermediate support status and previous psychological diseases. Depression was affected by marital status and social support, and its incidence increased significantly in patients who were widowed, divorced and only received moderate support. This suggests that we should pay more attention to the mental health of patients who receive low social support, those with previous psychological diseases, and those who are widowed or divorced.

According to the statistical analysis of this study, it was found that there was no significant difference in anxiety or depression among different genders, ages and education levels, which is contrary to the results of Mirzaei *et al*.^26^ The results of the study by Mirzaei *et al*. found that there was a significant difference between gender, age and education level in terms of anxiety or depression. Women were more anxious or depressed than men. As age and education decreased, the prevalence of anxiety or depression significantly increased, which may be related to the sample size, difference of grouping and cultural background of the selected subjects.

In a report by Ali *et al*. the anxiety and depression scores of patients with different social support states were different. Patients with lower social support were more likely to have anxiety and depression. Higher social support was beneficial to mental health, while the existence of lower social support was harmful to physical and mental health.^14^ Good social support can cushion stress and provide protection for individuals, and it is also important for maintaining a generally good emotional experience.^25^ This study showed that patients with previous psychological problems had higher anxiety scores, which may be due to the poor psychological quality of patients, low perception of life and the ability to deal with problems, and the more pressure they feel. This study showed that widowed and divorced patients had higher depression scores. The reason may be that a good marital relationship is an important factor to alleviate individual negative emotions and has an important impact on physical and mental health. In addition, illness can bring huge economic burden to patients and their families. Patients who are widowed or divorced are likely not to seek medical treatment, support and help actively, resulting in higher depression scores of patients.^26^

Pulmonary nodules have many psychological factors associated with anxiety and depression.^27^ The relationship between social support and anxiety and depression is complex. They affect and adjust each other, but the specific mechanism needs to be studied further.^28^ However, good psychological condition can help patients to relieve various stresses which affect their life and health, reduce the incidence of adverse events, improve their quality of life, thereby affecting their overall prognosis.^29^ It suggests that we should strengthen the communication between doctors and patients with pulmonary nodules, pay attention to the psychological status of such patients, apply the scale screening in time, diagnose and intervene anxiety and depression as soon as possible, and provide psychological counseling and other related measures if necessary. Only in this way can we promote the physical and mental health of patients, improve the effect of rehabilitation and treatment, avoid repeated medical treatment, reduce wastage of medical expenses, and improve the quality of life and social adaptability of patients.^30^

In conclusion, as the general use of chest CT scan including low‐dose computed tomography screening for lung cancer continues to grow, the risk of causing undue anxiety and depression becomes ever more present. In our study, anxiety was affected by social support and previous psychological illness, while depression was affected by marital status and social support. Poor psychological status is not conducive to the survival time and quality of life of patients, especially preoperative patients. More attention should be paid to some vulnerable groups, such as those with an unstable marriage, previous psychological diseases and those with low social support. At the same time, it is our duty to focus on the psychological status of such patients, strengthen communication, especially with preoperative patients, and as far as possible alleviate the negative feelings of patients in order to improve their prognosis and quality of life.

## Disclosure

The authors declare that there are no conflict of interest.
